# Delayed endovascular treatment of descending aorta stent graft collapse in a patient treated for post- traumatic aortic rupture: a case report

**DOI:** 10.1186/1749-8090-6-76

**Published:** 2011-05-24

**Authors:** Giovanni Nano, Daniela Mazzaccaro, Giovanni Malacrida, Maria Teresa Occhiuto, Silvia Stegher, Domenico G Tealdi

**Affiliations:** 1 University of Milan, Italy. 1st Unit of Vascular Surgery, IRCCS Policlinico San Donato, 20097 San Donato Milanese (MI), Italy

**Keywords:** TEVAR, traumatic aortic rupture, graft collapse

## Abstract

**Background:**

We report a case of delayed endovascular correction of graft collapse occurred after emergent Thoracic Endovascular Aortic Repair (TEVAR) for traumatic aortic isthmus rupture.

**Case presentation:**

In 7^th ^post-operative day after emergent TEVAR for traumatic aortic isthmus rupture (Gore TAG^® ^28-150), a partial collapse of the endoprosthesis at the descending tract occurred, with no signs of visceral ischemia. Considering patient's clinical conditions, the graft collapse wasn't treated at that time. When general conditions allowed reintervention, the patient refused any new treatment, so he was discharged.

Four months later the patient complainted for severe gluteal and sural claudication, erectile disfunction and abdominal angina; endovascular correction was performed. At 18 months the graft was still patent.

**Discussion and Conclusion:**

Graft collapse after TEVAR is a rare event, which should be detected and treated as soon as possible. Delayed correction of this complication can be lethal due to the risk of visceral ischemia and limbs loss.

## Introduction

Traumatic aortic isthmus rupture is the most common, life-threatening, thoracic aorta emergency in males in the fourth decade[1].

Endovascular technique is nowadays the gold standard treatment for this kind of lesion[2], but this approach may have some complications[3,4]. Graft collapse has been described in 33 cases in literature[5-18]; according to these reports, a reintervention is needed to correct this potentially lethal complication, either by open repair or with endovascular approach, as soon as the collapse is detected.

We report the case of an endovascular delayed repair of acute graft collapse occurred in a 40 years-old man who had been previously treated in emergency with TEVAR for traumatic isthmus rupture following a motocross accident.

## Case presentation

**Figure 1:**
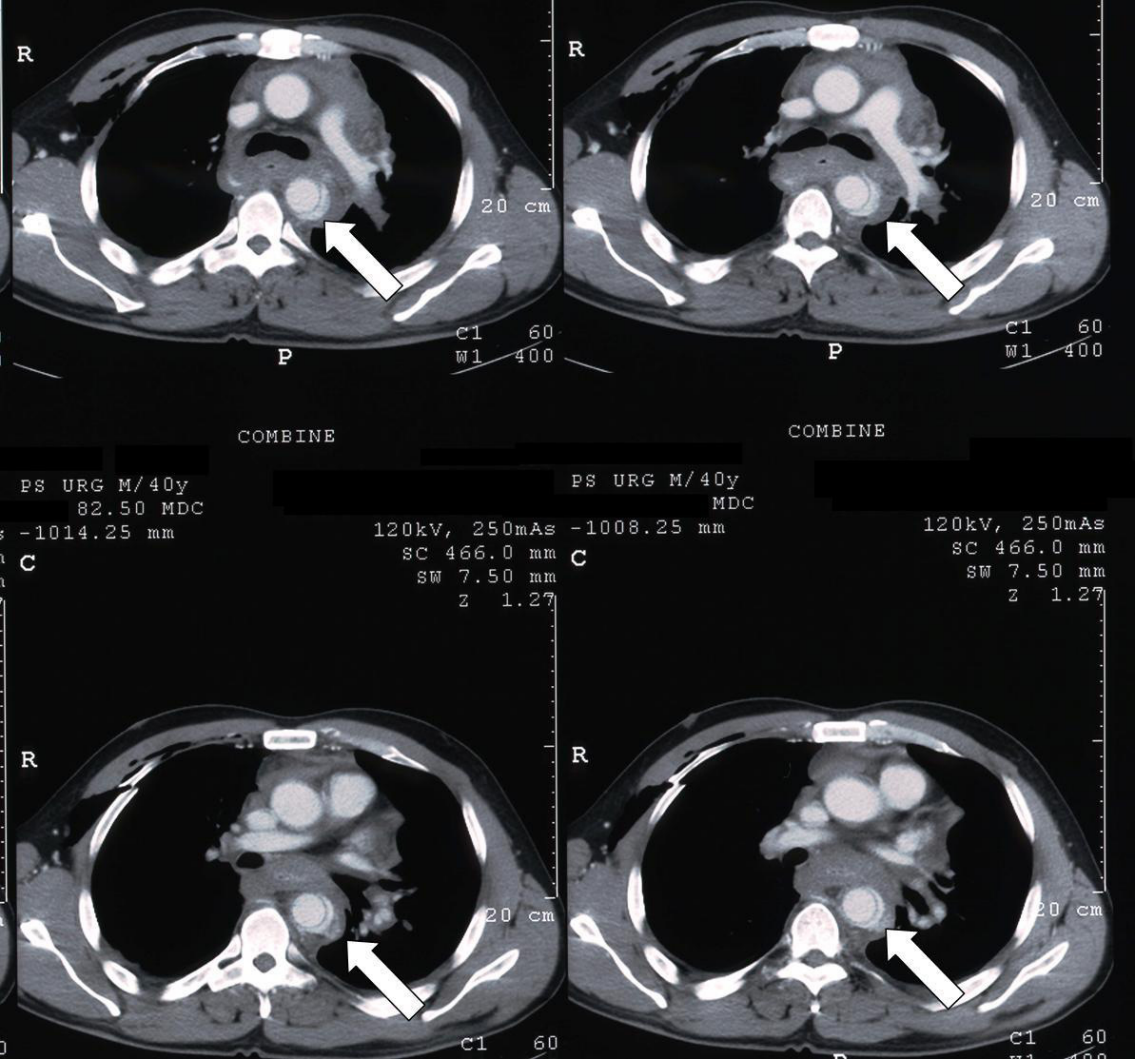
**Pre-operative CT scan**. Preoperative CT-scan showing isthmus rupture with dissection of the thoracic aorta.

A 40 years-old man was admitted to another hospital after a motocross accident. He had lung and liver contusion, right shoulder dislocation, multiple fractures of the right ribs and pneumo-hemothorax, and his blood pressure was 70/50 mmHg. A CT scan showed isthmus rupture with dissection of the thoracic aorta (Figure. 1). He was then transferred to our hospital, where he was immediately taken to operatory room to perform an endovascular correction of the lesion: an endoprosthesis (Gore TAG^® ^28-150, W.L. Gore and Associates, Flagstaff, Ariz) was placed from the distal tract of the aortic arch to the middle part of the descending aorta, with preservation of antegrade left subclavian artery flow. Intraoperative angiograms showed correct apposition of the graft to the vessel wall, without any signs of endoleaks. The patient was then admitted to the Intensive Care Unit for close monitoring and establishment of respiratory and hemodynamic stability. He didn't undergo any further surgical procedures for the treatment of other non-vascular lesions.

**Figure 2:**
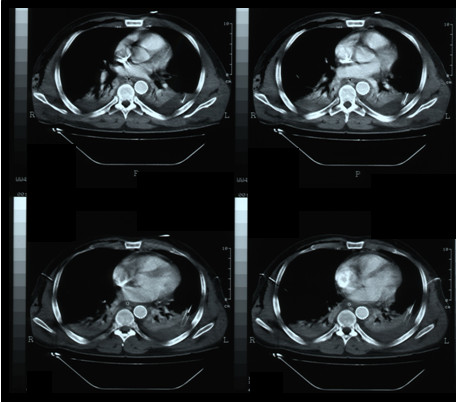
**CT scan at 2^nd ^postoperative day**. The images show good apposition of the graft to the aortic wall, with no signs of endoleak.

Two days later a second CT scan was performed to control the correct placement of the graft, since the patient's hemodynamic condition was still unstable. The images showed right lung emphysema, enlargement of mediastinum, pericardial effusion, bilateral pleural effusions with pulmonary atelectasy due to compression of the parenchyma and an increase of the haematoma surrounding the aortic arch and subclavian artery; there weren't any signs of endoleak nor further bleeding (Figure. 2).

**Figure 3:**
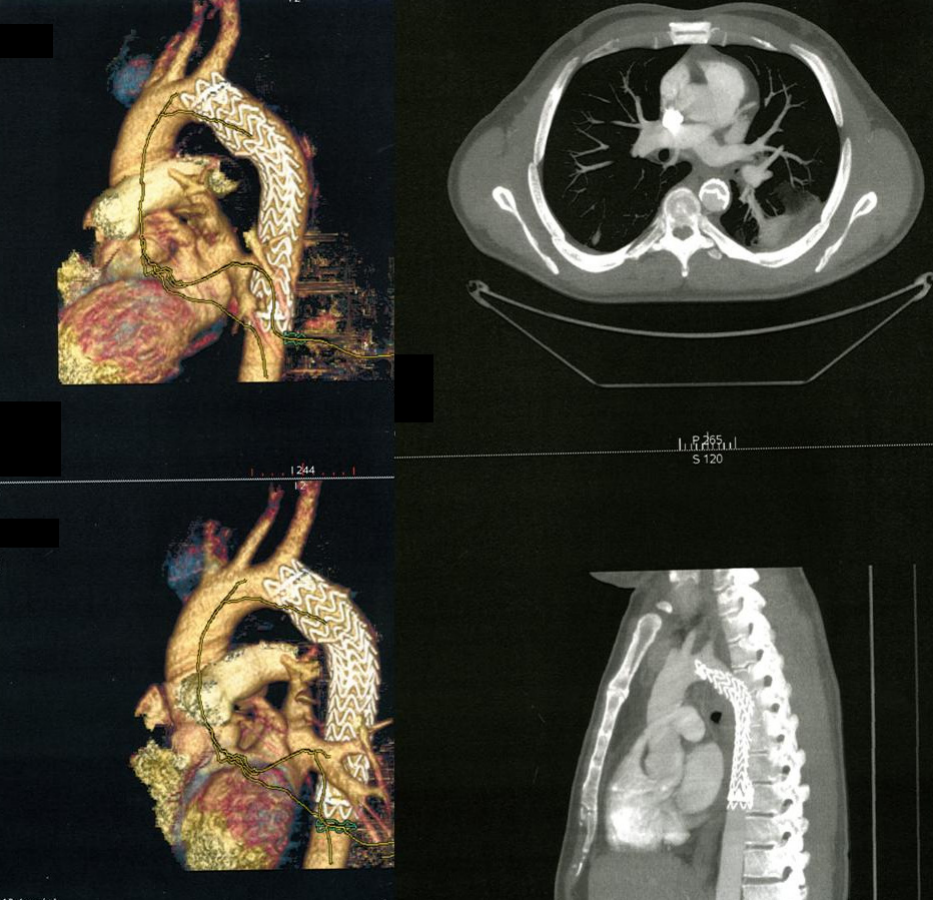
**CT scan before discharge**. Partial collapse of the endoprosthesis at the descending tract.

On the following week signs of aortic pseudocoarctation syndrome occurred, with no popliteal and tibial pulses; femoral pulses were decreased but still palpable. A new CT scan was then performed in 7^th ^post-operative day: no signs of graft rupture were evident but there was a partial collapse of the endoprosthesis at the descending tract, with distal slow restoration of the blood flow throughout the right lumen (Figure. 3); the patient still had pulmonary atelectasis due to hemotorax and ribs fractures.

Considering patient's clinical conditions, the graft collapse wasn't treated at that time, waiting for hemodynamic stability. Since then, the patient's general conditions progressively improved enough to allow reintervention, but the patient refused any new treatment, so he was discharged.

**Figure 4:**
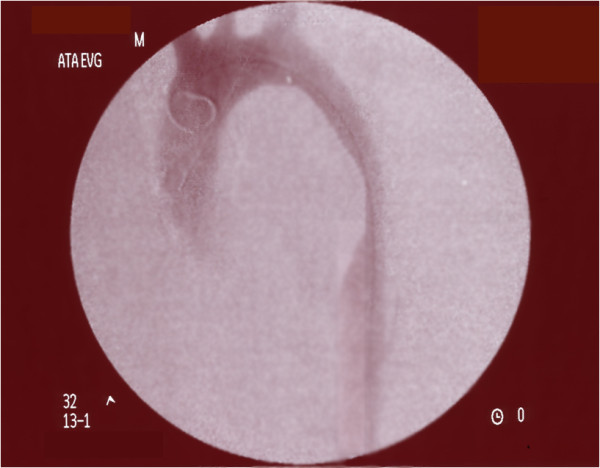
**Angiogram during reintervention**. The angiogram demonstrates the increased collapse of the aortic graft with a sub-occlusion of its lumen.

Four months after the procedure the patient came back to our Division. He complained for gluteal and sural claudication with pain-free walking interval of less than 40 meters and erectile disfunction; a chest and abdominal radiography showed a subocclusion of the graft. A ultrasound duplex examination of abdominal aorta and lower limb arteries showed revascularization flows throughout splancnic vessels and both common, internal and external iliac arteries. We thus decided to perform an angiography which demonstrated the increased collapse of the aortic graft, confirming the sub-occlusion of the central tract of its lumen, with a delayed flow in the abdominal aorta (Figure. 4).

**Figure 5:**
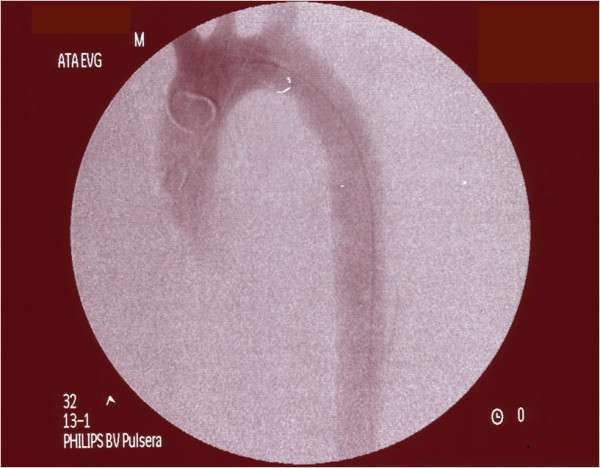
**Correction of the lesion**. A Bolton Relay™ 28-155 graft located inside the previous one, with a bare-stent at the left subclavian artery, restores a single inner lumen and allows a continuous and valid blood flow to the descending aorta.

A second graft (Bolton Relay™ 28-155, Bolton Medical, Barcelona, Spain) was then placed inside the previous one, with a bare-stent which landed proximally to the left subclavian artery (Figure. 5). As there were some difficulties in ascending the guidewire across the collapsed graft, it was ballon-dilated before introducing the new endograft. No post-dilatation was necessary. A single inner lumen was restored to allow a continuous and valid blood flow to the descending aorta. The patient was discharged four days later in good clinical conditions. Peripheral pulses were all presents and palpable.

**Figure 6:**
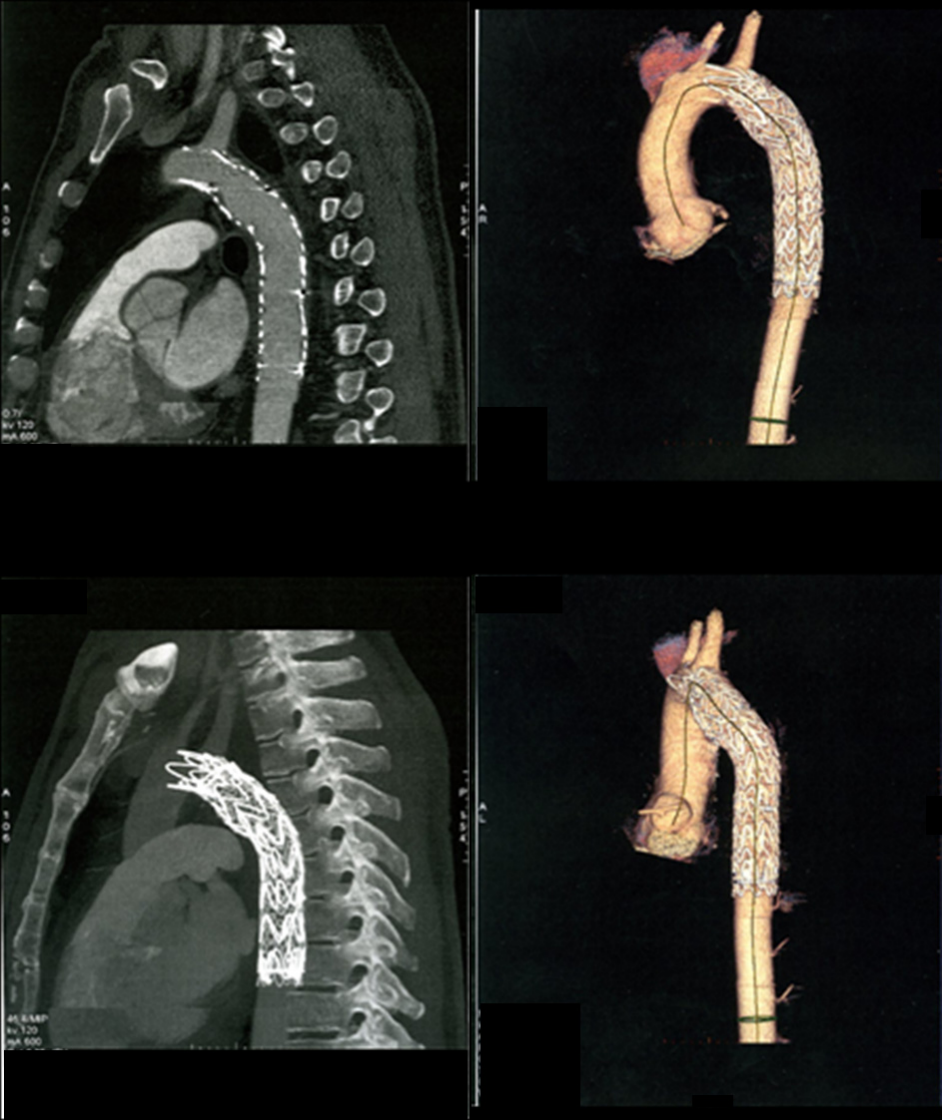
**CT scan at 18 months**. Regular diameter of the graft, normal renal perfusion, no signs of any endoleaks.

At the 18th month follow-up a CT scan showed regular diameter of the graft, normal renal perfusion, no signs of any endoleaks; the left subclavian artery remained patent at follow-up (Figure. 6). The patient returned to his previous daily activities; he didn't complain of claudication anymore and its erectile function had returned to normal.

## Discussion and conclusion

There is no doubt that immediate diagnosis and treatment are mandatory to reduce mortality of traumatic thoracic aortic ruptures[19].

Since 1992 endovascular stent technology has been proposed as a valid alternative to surgery in the treatment of injured thoracic aorta[20]; endovascular repair represents a less invasive therapy for the treatment of all kinds of aortic diseases, including urgent management of polytraumatic patients affected by aortic dissection. Moreover, it carries encouraging early and medium- term outcomes[19].

Anyway it carries several possible complications[3]; in addition, endograft collapse was described mostly after TEVAR for traumatic aortic rupture[5].

Review of the literature suggests that the timing of collapse is highly variable and can occur from 3 days to 11 months[14]. It seemed to be significantly related with small (<23 mm) aortic diameters and second generation TAG device[5]. Current grafts in fact are designed to treat atherosclerotic diseases and they present greater diameters. An excessive oversizing, greater than 25% may cause wrinkling of the graft with a lack of apposition to the aortic wall. Moreover young patients present often acute angulated aortic necks with smaller radius of the lesser curvature of the aortic arch; this may represent an inadequate proximal landing zone for current grafts which have limited conformability and longitudinal flexibility.

In our case, the proximal aortic diameter was 21 mm and we used a 28 mm graft, with an oversizing of 33%. Probably this high oversizing was one of the causes of endograft collapse. Our main aim was however to save the patient's life, as the patient needed an emergent treatment; for logistic reasons the oversized stent grafts were the only type available in an emergency setting, and we didn't have the time to wait for the right graft. If we had had time we would have placed a smaller Bolton Relay graft immediately.

Moreover, to minimize the risk of vertebro-basilar insufficiency and paraplegia, we chose not to cover the patient's left subclavian artery, even if it would have ensured a better and longer proximal landing site. Young patients, in fact, are at risk of left arm claudication, even though it has been shown that covering of the left subclavian artery can be performed without significant morbidity[21].

Natural history of stent-graft collapse is not fully understood; for this reason, all cases that are reported in literature were treated as soon as they were detected. To our knowledge, this report is the first case of delayed repair of graft collapse; the delay was due to the comorbid conditions and the need to establish hemodynamic stability. Moreover the patient didn't show any signs of visceral nor critical lower limb ischemia, so we opted for a strict surveillance until general condition could have improved and any new intervention could have been performed.

Unfortunately, when general conditions had improved enough to allow of a reintervention, the patient refused any new treatment. We feared the occurrence of lethal complications, such as visceral ischemia, and the impossibility to perform the future correction through endovascular approach: we aimed to avoid a thoracotomy in a patient who was recovering from rib fractures, pneumo-hemothorax and lung contusion.

Four months later, symptoms had become disabling, so the patient decided to undergo the reintervention; correction of the lesion was then performed using an additional endoprosthesis plus a bare stent deployed inside the collapsed graft, slightly proximally, with good clinical and technical results. We didn't use a single stent or a single endograft because we wanted to ensure a better proximal landing zone leaving the left subclavian artery still patent.

Discussion is still open about the best treatment of this kind of complications, especially in young patients. Nobody knows the potential risk of aortoesophageal fistula[13].

In conclusion, TEVAR has significantly improved the treatment of thoracic aortic rupture. Graft collapse is a rare event, which should be detected and treated as soon as possible. Delayed correction of this complication can be lethal due to the risk of visceral ischemia and limbs loss.

Further technical modifications of devices are necessary in order to minimize complications and improve clinical outcomes.

## Consent

Written informed consent was obtained from the patient for publication of this case report and accompanying images. A copy of the written consent is available for review by the Editor-in-Chief of this journal.

## Competing interests

The authors declare that they have no competing interests.

## Authors' contributions

*GN designed the case report and performed the search in the literature*.

*DM participated in the design of the report and performed the search in the literature*.

*GM, MTO, SS, DGT participated in the design and coordination of the report*.

*All authors read and approved the final manuscript*.

## IRB Approval

*Our institution approved the report of this case*.
